# A gallbladder tumor revealing metastatic clear cell renal carcinoma: report of case and review of literature

**DOI:** 10.1186/1746-1596-8-4

**Published:** 2013-01-10

**Authors:** Merieme Ghaouti, Kaoutar Znati, Ahmed Jahid, Fouad Zouaidia, Zakiya Bernoussi, Youssef El Fakir, Najat Mahassini

**Affiliations:** 1 Department of Pathology, Ibn Sina Univesity Hospital, Rabat, Morocco; 2 Nakhil Radiology Center, Rabat, Morocco

**Keywords:** Kidney tumors, Clear cell renal cell carcinoma, Gallbladder metastasis, Cholecystectomy

## Abstract

Metastatic renal cell carcinoma in the gallbladder is extremely rare, with reported frequencies of less than 0.6% in large autopsy reviews. Only 40 cases were reported in the literature. We report a first case of gallbladder polypoid tumor revealing metastatic clear cell renal cell carcinoma, which demonstrates the importance of radiological tests, histology and immunohistochemistry when making a definitive diagnosis. These examinations also allow differentiating metastatic clear cell renal cell carcinoma from other polypoid lesions in the gallbladder with clear cell morphology. Cholecystectomy should be performed to obtain a definitive diagnosis and to improve survival in case of solitary metastatic renal cell carcinoma.

**Virtual slides:**

The virtual slides’ for this article can be found here: http://www.diagnosticpathology.diagnomx.eu/vs/8956897238238989

## Background

Metastatic tumors to the gallbladder are uncommon. The most common metastatic tumors to the gallbladder are metastatic melanomas and metastatic carcinomas from stomach, pancreas, ovary, bile ducts, colon and breast [1]. Metastatic renal cell carcinoma in the gallbladder is extremely rare, with reported frequencies of less than 0.6% in large autopsy reviews [2]. Renal cell carcinoma is a rare tumor accounting for 3% of all malignancies in adults and 85% of primary renal tumors. However, this tumor has a great propensity for metastasizing synchronously or metachronously to various anatomic sites [3]. We report a case of intraluminal polypoid metastasis of clear cell renal cell carcinoma in gallbladder mimicking gallbladder polyp and revealing the renal carcinoma and reviewed the reported 40 cases. The clinico-pathologic features and differential diagnosis are discussed.

## Case presentation

### Case report

**Figure 1:**
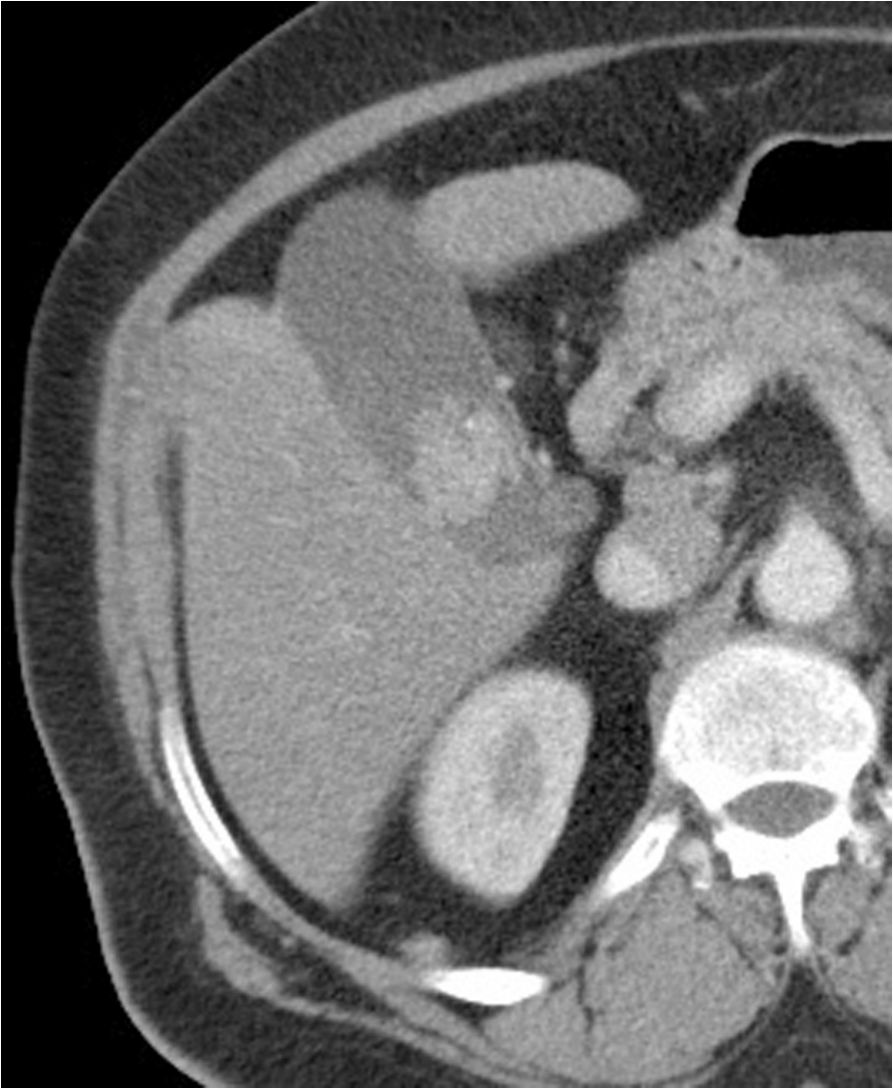
Axial computed tomography image after intravenous contrast enhancement reveals an intraluminal pedunculated hyperdense enhancing mass in the gallbladder without thickening of the gallbladder wall.

**Figure 2:**
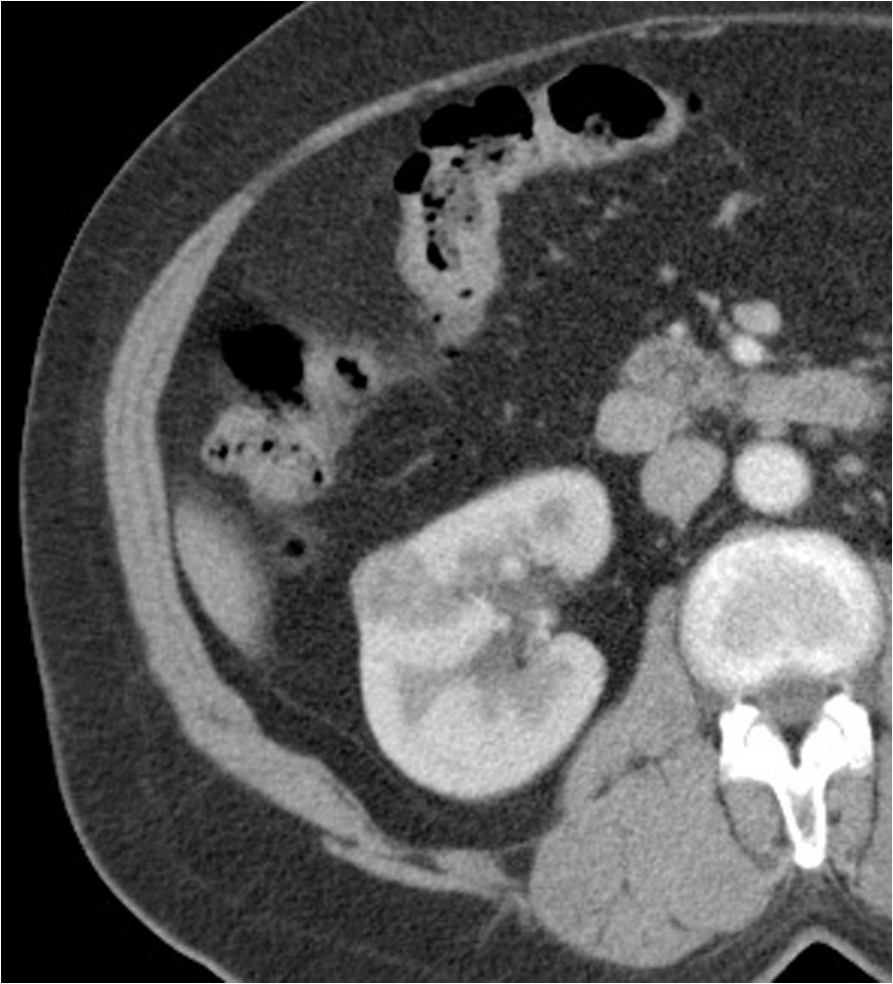
Axial computed tomography image after intravenous contrast enhancement shows a cortical fleshy nodule of the right kidney with no involvement of the perinephric adipose.

A 55-year-old woman presented with severe right hypochondrium pain, with weight loss and alteration of her general condition, lasting for 6 months. She showed no urological signs, especially no hematuria, no pain, and no flank mass. She had no past history in particular. Neither physical examination nor laboratory examination revealed any significant findings. Abdominal ultrasonography showed a 2.6 cm diameter intraluminal polypoid hyperechoic mass in the gallbladder. Color Doppler ultrasonography examination demonstrated vessels in the mid-portion of the mass. Computed tomography scan confirmed the presence of a gallbladder tumor that appeared as an enhancing pedunculated tumor within the gallbladder, without thickening of the gallbladder wall. The tumor seemed to be attached to the edge of the liver with no macroscopic extension to the liver parenchyma (Figure 1). Furthermore, TDM scan showed a cortical fleshy nodule of the right kidney, measuring 2.2 cm, with no involvement of the perinephric adipose (Figure 2). The biliary symptoms and pedunculated appearance of the tumor on the TDM scan suggested a possible diagnosis of primary carcinoma of the gallbladder. Because of the small size of the renal nodule and the absence of urological signs, a close monitoring of the renal lesion with a control after 6 months was first advocated. The patient underwent an open cholecystectomy with a partial liver resection.

**Figure 3:**
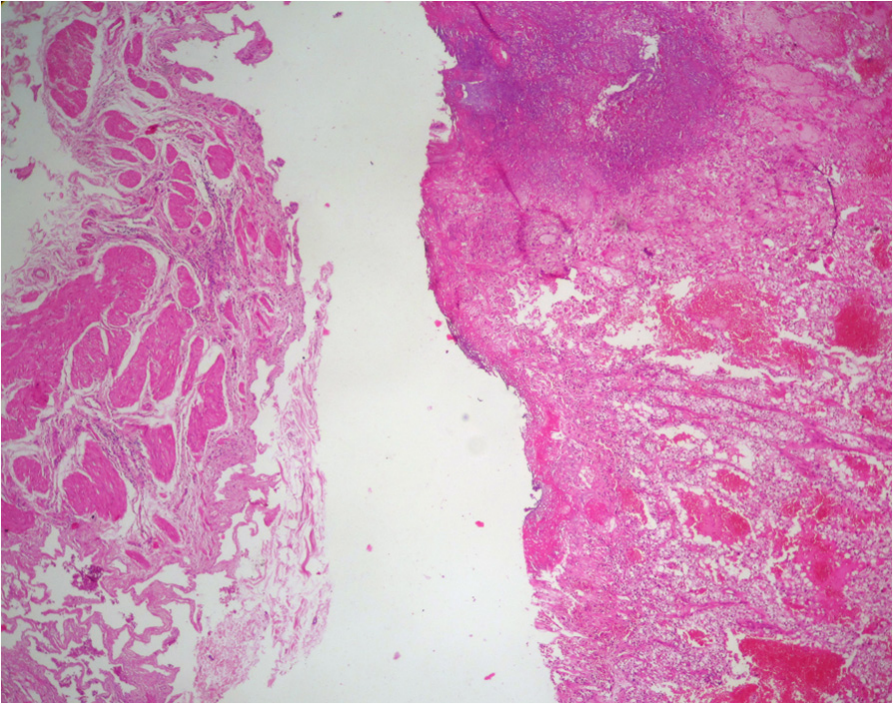
Representative micrograph shows that the polyp is composed of clear cell renal cell carcinoma in a background of normal gallbladder (Hematoxylin-eosin, original magnification x4).

**Figure 4:**
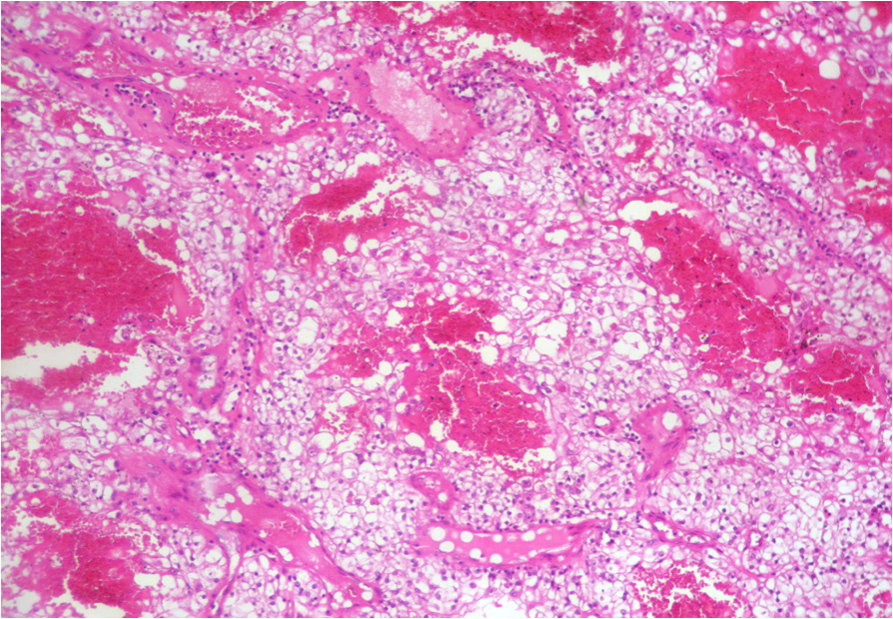
**Representative micrograph of the polyp: The tumor is composed of clear cells disposed in alveolar pattern with delicate arborizing capillary vascular network.** (Hematoxylin-eosin, original magnification x10).

**Figure 5:**
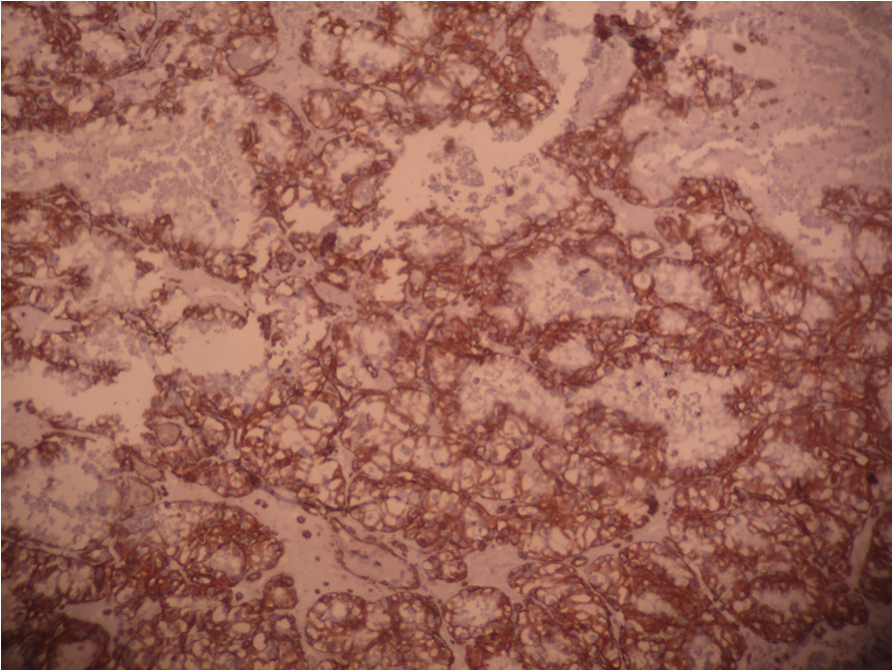
Immunohistochemical stain: The tumor cells are positive for CD10.

**Figure 6:**
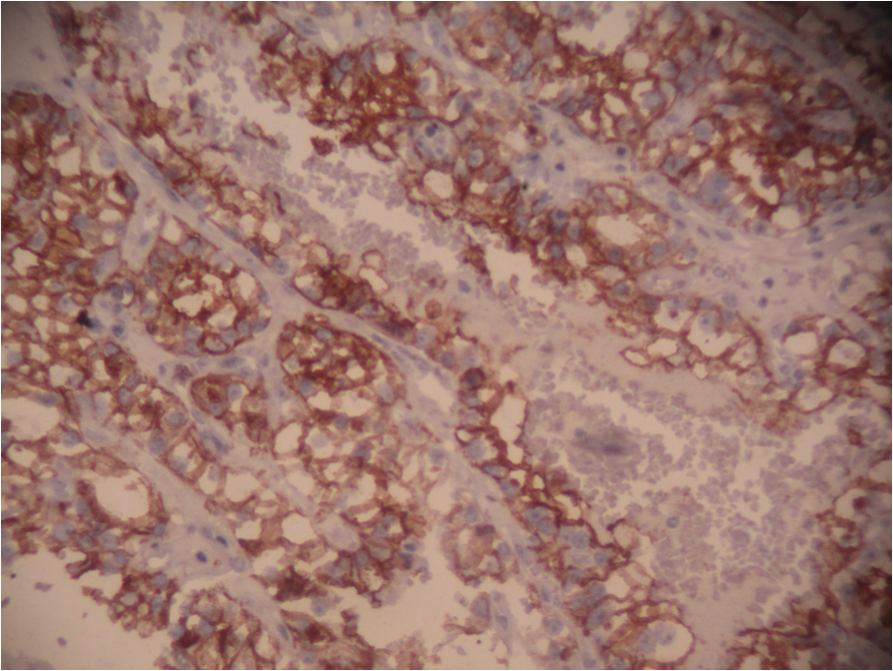
Immunohistochemical stain: The tumor cells are positive for vimentin.

The cholecystectomy specimen had a 4.0 × 3.0 × 2.0 cm, dislodged polypoid mass free floating in the lumen of the gallbladder. The surface of the mass was covered by bile and necrotic debris. Cut surface of the mass was homogenous, yellow-tan and soft, with foci of hemorrhage and necrosis. The gallbladder mucosa was pale-tan and slightly thickened. Grossly, no involvement of the gallbladder wall was identified. Gallstones were not found in the bile. Histologically, the polyp consisted of clear cells with delicate arborizing capillary vascular network, consistent with a metastatic conventional clear cell renal cell carcinoma (Figure 3). Tumor cells were disposed in solid and alveolar patterns. The cytoplasm was abundant, clear, and surrounded by a distinct cell membrane. The nucleus was round and uniform, with finely granular, evenly distributed chromatin (Figure 4). There was no apparent involvement of the gallbladder mucosa or the gallbladder wall. The overlying mucosa showed no atypia or dysplasia. The cystic duct resection margin was negative for carcinoma. The liver parenchyma was not invaded by the carcinoma. Diagnosis of metastatic renal cell carcinoma (RCC) was confirmed by immunohistochemical stains, which showed the tumor cells to be positive for CD10 (Figure 5), vimentin (Figure 6) and pancytokeratin, but negative for CK7, carcinoembryonic antigen (CEA), chromogranin, synaptophysin and CD68.

**Figure 7:**
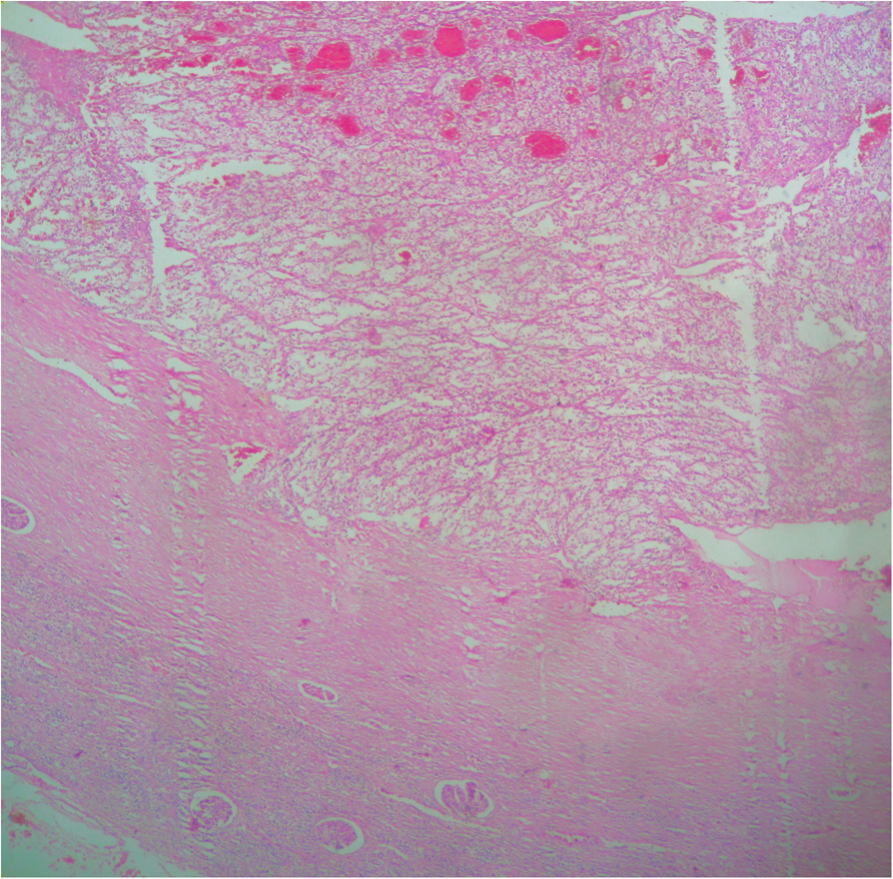
Clear cell renal cell carcinoma of the kidney (Hematoxylin-eosin, original magnification x4).

In light of these new data, the patient was transferred to the department of Urology for a possible nephrectomy. A right radical nephrectomy was carried out through right subcostal incision without demonstrable vascular invasion or involvement of the perinephric adipose tissue intraoperatively. On gross examination, the nephrectomy specimen showed a 2.2 cm yellowish cortical solid mass with foci of hemorrhage. The histological study revealed a typical conventional clear cell RCC, Fuhrman nuclear grade 3, with negative surgical resection margins (Figure 7).

## Discussion

Renal cell carcinomas (RCCs) represents on average over 90% of all malignancies of the kidney. Approximately 20%-30% of patients with RCC have metastatic disease at presentation and nearly 50% of patients with advanced disease die within 5 years of diagnosis. Global incidence of RCC continues to grow steadily with the increase in incidentally discovered lesions during imaging studies. Up to 50%-60% of RCCs may be incidentally found in asymptomatic patients at abdominal imaging studies performed for other indications [4]. Recent advances in the understanding of the genetic basis of RCC have provided unique insights into the underlying histologic and biological diversity of renal cancer. Renal cell carcinoma is considered a byproduct of genetic events that may involve gain-of-function of proto-oncogenes, loss-of-function of cancer suppressor genes, or both [5]. Different histologic subtypes of renal cell carcinoma have characteristic clinical, genetic, and biological profiles. Indeed, the 2004 World Health Organization recognizes that RCC is a clinicopathologically heterogeneous malignant neoplasm that can be classified into clear cell, papillary, chromophobe, collecting duct, medullary carcinoma, and unclassified categories. Clear cell RCC is the most common histologic subtype representing 70%-75% of all RCCs. Papillary RCC and chromophobe RCC account for 15%-20% of the remainder of the RCCs [6]. Distinct differences in biological behavior and long-term prognosis among different subtypes of RCC makes the correct histologic diagnosis critically important. Clear cell RCC is characterized by glycogen and lipid-rich clear cells within a regular network of small, thin-walled blood vessels. Papillary RCC typically consists of papillae containing a delicate fibrovascular core and covered by epithelial cells. Chromophobe RCCs are histologically characterized by large polygonal pale cells with prominent cell membranes and perinuclear clearing [6].

Clear cell RCC is characterized by its great propensity to metastasize synchronously or metachronously to various anatomic sites. Clear cell RCCs most commonly metastasize hematogenously via the vena cava primarily to the lung, although lymphatic metastases also occur. Retrograde metastasis along the paravertebral veins, the vena testicularis/vena ovarii, intra-renal veins, or along the ureter may also occur [6]. The most common sites of metastasis are the lung, bone, brain, liver, adrenal gland, the other kidney, and rarely, organs such as the vertebrae, stomach, spleen, pancreas, and diaphragm [7]. Clear cell RCC is well known for its propensity to metastasize to unusual sites. Metastatic renal cell carcinoma in gallbladder is extremely rare. Indeed, the gallbladder was recognized as a site of metastasis in only 4 out 687 cases (0.58%) in large autopsy reviews [2]. Furthermore, metastatic gallbladder lesions are infrequent; melanoma, stomach, pancreas, ovary, canal bowel, biliary duct and breast carcinomas are those that have the highest probability of metastasis. Half of the cases are synchronic [8]. To our knowledge, only 40 cases of metastatic renal cell carcinoma in gallbladder were reported in the literature.

Clinical findings are not specific enough to arrive at a final diagnosis. Metastatic tumors in gallbladder usually appear in 39 to 84-year-old-males, who have a personal history of cancer without previous biliary pathology, while primary tumors prevail in women over 65 years of age with biliary lithiasis [9]. In case of synchronous gallbladder metastasis, the primary renal cell cancer is found by TDM-scan who shows both lesions. However, imaging diagnosis may not be conclusive in making a differential diagnosis between primary and secondary tumors. Ultrasonography is the initial approach in the diagnosis of gallbladder tumors. Metastases can appear under different hyperechoic masses bigger than 1 cm, close to the gallbladder wall without posterior echogenic shadowing [10]. Primary tumors appear as solid mass occupying the entire wall thickness, or as a polypoid lesion with increased vascularization [10]. Invasion of the mucosal layer is another radiological finding that can help to differentiate between a primary gallbladder tumor and metastasis in the computed tomography scan. If the mucosa is not infiltrated, indicating an invasion from the serosa layer, the primary gallbladder tumor can be excluded [10].

The pathologic study is the key of diagnosis. Macroscopically, reported metastatic renal cell carcinoma of the gallbladder showed a single, intraluminal, polypoid or pedunculated mass. In one reported case, like in our case, the metastatic renal cell carcinoma was a free-floating polyp within the lumen of the gallbladder with no apparent involvement of the gallbladder wall [8]. The clear cell RCC is typically golden yellow on cut surface, due to the rich lipid content of its cells. Necrosis and hemorrhage are commonly present. Microscopically, almost all cases showed clear cell renal carcinoma [8]. Clear cell RCC is architecturally diverse, with solid, alveolar and acinar pattern, the most common. The carcinomas typically contain a regular network of small thin-walled blood vessels, a diagnostically helpful characteristic of this tumor. The alveolar and acinar structures may dilate, producing microcystic and macrocystic patterns. Tumor cells have an abundant clear cytoplasm surrounded by a distinct cell membrane. High grade tumors may contain minority populations of cells with eosinophilic cytoplasm. The nuclei tend to be round or uniform with finely granular, evenly distributed chromatin. Depending upon the grade, nucleoli may be inconspicuous, small, or large and prominent. Most clear cell RCCs have little associated inflammatory response [6]. Metastatic clear cell RCC in gallbladder is confirmed by immunohistochemistry, using vimentin and CD10 in particular. In our case, the tumor cells were positive for vimentin, CD10, and pancytokeratin, but negative for CK7, CEA, chromogranin, synaptophysin and CD68.

The specific diagnosis of polypoid gallbladder masses is problematic. Because of the morphologic characteristics of metastatic clear cell RCC in gallbladder, this tumor should be distinguished from other polypoid lesions in the gallbladder with clear cell morphology, including clear cell carcinoma of gallbladder, clear cell carcinoid tumors, paragangliomas and cholesterol polyps. Clear cell morphology is identical in both metastatic clear cell RCC and clear cell carcinoma of gallbladder. Indeed, clear cell carcinomas of the gallbladder are composed of clear cells arranged in glands, sheets, nests, trabeculae and papillary structures. The predominance in women and the presence of gallstones, mucin and other components, including conventional adenocarcinomas, or squamous differentiation in clear cell carcinoma helps depart a primitive clear cell carcinoma of gallbladder [9]. However, in some cases, only immunohistochemical study can establish with certainty the primitive or metastatic character of carcinoma. Indeed, while some markers like broad spectrum cytokeratin and epithelial membrane antigen (EMA) are expressed by both tumor types, others are specific to one or the other. Thus, vimentin and CD10 are expressed only in clear cell renal cell carcinoma; CEA is expressed only in gallbladder carcinomas [9]. In our case, tumor cells expressed vimentin and CD10. Immunostaining with CEA was negative. Metastatic clear cell RCC should also be differentiated from a clear cell carcinoid tumor of the gallbladder. In the latter, tumor cells arrange in nests and tubules within a delicate capillary vascular network. However, they typically invade through the muscular layer into perimuscular soft tissue. Immunohistochemical stains are helpful in differentiating metastatic clear cell RCC from clear cell carcinoid tumor of the gallbladder. The latter is positive for neuroendocrine markers. Metastatic clear cell renal cell carcinoma and clear cell carcinoma of the gallbladder are negative for neuroendocrine markers [8]. Paraganglioma is extremely rare in gallbladder. It appears as a small, well-circumscribed nodule protruding to the external surface of the gallbladder. The tumor is composed of chief and sustentacular cells arranged in a zellballen pattern. Furthermore, tumor cells in paraganglioma are positive for neuroendocrine markers with positive staining for S100 in the sustentacular cells [1]. Cholesterol polyps are small, yellow, multilobulated polyps attached to the gallbladder mucosa by a thin pedicle. Histologically, the tumor is composed of foamy histiocytes covered by gallbladder mucosa and lack the delicate arborizing capillary network seen in clear cell renal carcinoma. These histiocytes are positive for oil-red O and CD68 but negative for epithelial markers by immunohistochemistry [1].

In all gallbladder findings with suspected malignancy, or benign lesions larger than 1 cm, a cholecystectomy should be performed to obtain a definitive diagnosis [11]. Cholecystectomy with R0 resection has been demonstrated to be the only factor that increases survival mainly in isolated cases of metastasis [8]. Synchronous metastasis of RCC in the gallbladder at the time of nephrectomy does not correlate with poor prognosis. Acute cholecystis as a clinical presentation is associated with poor prognosis [12]. The five-year survival rate following cholecystectomy for RCC is 35-50%. According to Chung et al. [11], 63% of patients with one gallbladder metastasis have a two-year survival, while in the case of multiple metastases this rate decreases to 23% following cholecystectomy.

Last case reports showed that solitary metastasis of renal cell carcinoma in the gallbladder without evidence of involvement of other anatomic sites correlated with better survival [8]. Simple cholecystectomy for metastatic renal cell carcinoma may provide favorable long-term survival of patients with renal cell carcinoma [11].

## Conclusion

In summary, we report a case of a free-floating intraluminal polyp of the gallbladder consistent with a metastatic clear cell RCC and reviewed the 40 published cases on metastatic renal cell carcinoma in the gallbladder in the literature. This is the only case in which the renal cell carcinoma was revealed by its metastasis in the gallbladder. The importance of histological and, especially, immunohistochemical studies had been proven in distinguishing metastatic clear cell carcinoma in the gallbladder from other polypoid lesions of gallbladder with clear cell morphology. This distinction was crucial for an optimal management of both the gallbladder tumor and the primitive renal carcinoma.

## Consent

Written informed consent was obtained from the patient for publication of this case report and accompanying images. A copy of the written consent is available for review by the Editor-in-Chief of this journal.

## Abbreviations

RCC: Renal cell carcinoma; CEA: Carcinoembryonic antigen; EMA: Epithelial membrane antigen.

## Competing interests

The authors declare that they have no competing interests.

## Authors’ contribution

MG retrieved clinical information, wrote the manuscript and performed the literature review. KZ first identified this case, proposed the study and revised the manuscript for important intellectual content. AJ and YEF acquired photomicrographs. FZ, ZB and NM provided valuable insight during manuscript preparation. All authors read and approved the final manuscript.
